# Cost-Effectiveness Analysis of Routine Use of 15-Valent Pneumococcal Conjugate Vaccine in the US Pediatric Population

**DOI:** 10.3390/vaccines11010135

**Published:** 2023-01-06

**Authors:** Min Huang, Tianyan Hu, Jessica Weaver, Kwame Owusu-Edusei, Elamin Elbasha

**Affiliations:** Merck & Co., Inc., Rahway, NJ 07065, USA

**Keywords:** cost-effectiveness, PCV15, V114, pneumococcal diseases, pediatric vaccination, public health impact

## Abstract

This study evaluated the clinical and economic impact of routine pediatric vaccination with the 15-valent pneumococcal conjugate vaccine (PCV15, V114) compared with the 13-valent PCV (PCV13) from a societal perspective in the United States (US). A Markov decision-analytic model was constructed to estimate the outcomes for the entire US population over a 100-year time horizon. The model estimated the impact of V114 versus PCV13 on pneumococcal disease (PD) incidence, post meningitis sequalae, and deaths, taking herd immunity effects into account. V114 effectiveness was extrapolated from the observed PCV13 data and PCV7 clinical trials. Costs (2021$) included vaccine acquisition and administration costs, direct medical costs for PD treatment, direct non-medical costs, and indirect costs, and were discounted at 3% per year. In the base case, V114 prevented 185,711 additional invasive pneumococcal disease, 987,727 all-cause pneumonia, and 11.2 million pneumococcal acute otitis media cases, compared with PCV13. This led to expected gains of 90,026 life years and 96,056 quality-adjusted life years with a total saving of $10.8 billion. Sensitivity analysis showed consistent results over plausible values of key model inputs and assumptions. The findings suggest that V114 is a cost-saving option compared to PCV13 in the routine pediatric vaccination program.

## 1. Introduction

*Streptococcus pneumoniae* (*S. pneumoniae,* pneumococcus) is an important pathogen associated with considerable disease burden across all ages, with young children and elderly adults being most vulnerable [1,2,3]. This pathogen can lead to invasive pneumococcal disease (IPD), such as meningitis, bacteremia, bacteremic pneumonia and sepsis, with substantial morbidity and mortality [4,5,6]. More commonly, pneumococcus causes non-invasive pneumococcal diseases (PDs), including pneumonia, acute otitis media (AOM) and sinusitis [4,6]. Among these diseases, meningitis is the most serious form associated with a high mortality and long-term disabling neurological sequelae, such as hearing loss, seizures, and cognitive deficit, despite optimal treatment [5,7,8]. Both IPD and non-invasive PD are associated with substantial economic burden, including direct and indirect costs that are related to productivity loss and premature death [9,10,11,12].

Before the introduction of pneumococcal vaccines in routine immunization schedules, the incidence of IPD was about 95 cases per 100,000 in children <5 years old in 1998 in the United States (US) [13]. The disease burdens of pneumococcal pneumonia and AOM, for which pneumococcus was the most common cause, were also high at that time [14,15]. In 2000, the Centers for Disease Control and Prevention (CDC) Advisory Committee for Immunization Practices (ACIP) recommended the 7-valent pneumococcal conjugate vaccine (PCV7) be administered at 2, 4, 6 and 12–15 months for all children aged 2–23 months. It was also recommended for high-risk children of 24–59 months [4]. The recommendations marked the beginning of universal routine immunization of infants and toddlers with PCVs in US and worldwide. In 2010, with the approval of 13-valent PCV (PCV13), the ACIP updated the recommendations with routine use of PCV13 instead of PCV7 [16]. Since the advent of PCVs more than 20 years ago, a reduction of 91% of IPD cases and 41% of annual AOM visits was observed in children <5 years in the US [17]. In addition, a recent review has shown significant indirect effect (i.e., herd immunity) of PCVs in multiple global studies, with a median of 57% reduction in vaccine-type IPD cases [18].

Despite the success in the prevention of PD following the introduction of PCVs, substantial residual disease caused by non-vaccine types (NVTs) remains. In 2017–2018, non-PCV13 serotypes accounted for 77% of IPD cases among children <5 years in the US [19]. In addition, certain PCV13 serotypes persist, such as serotype 3 [20,21,22,23]. There is a need for expanded valency PCVs that maintain the protection against disease causing vaccine-type serotypes, while adding protection against disease caused by NVT serotypes.

Recently, the Food and Drug Administration (FDA) expanded the indication of a 15-valent PCV, (PCV15, V114, VAXNEUVANCE™, Merck Sharp & Dohme LLC, a subsidiary of Merck & Co., Inc., Rahway, NJ, USA) from adults to active immunization for IPD prevention in children aged 6 weeks and older [24]. Similarly, the European Commission also expanded V114′s indication for the prevention of IPD, pneumonia and AOM caused by *S. pneumoniae* in individuals from 6 weeks to less than 18 years of age [25]. V114 contains all serotypes included in PCV13 (i.e., 1, 3, 4, 5, 6A, 6B, 7F, 9V, 14, 18C, 19F, 19A, and 23F) and two additional serotypes, 22F and 33F. These two serotypes accounted for 17% of all IPD cases among children <5 years in the US in 2017 [26] and are associated with high mortality, antibiotic resistance and meningitis [2]. In clinical trials in children, V114 was well-tolerated and demonstrated a comparable safety profile to PCV13; immune responses were comparable to PCV13 for the 13 shared serotypes [24,27,28]. Additionally, the pivotal phase 3 pediatric trial demonstrated statistically superior immune responses in V114 for serotypes 3, 22F and 33F, compared to PCV13 [28].

Following the FDA’s approval, the ACIP updated the recommendations on pneumococcal vaccines for pediatric use in the US. Specifically, it recommends V114 as an option for vaccination in children < 19 years old, including routine use in children aged <2 years [29]. The recommendations were based on evidence of the immunogenicity, safety and cost-effectiveness of V114. The current study provided the details on the cost-effectiveness of V114 versus PCV13 in the pediatric population from a societal perspective in the US.

## 2. Methods

### 2.1. Model Overview

**Figure 1:**
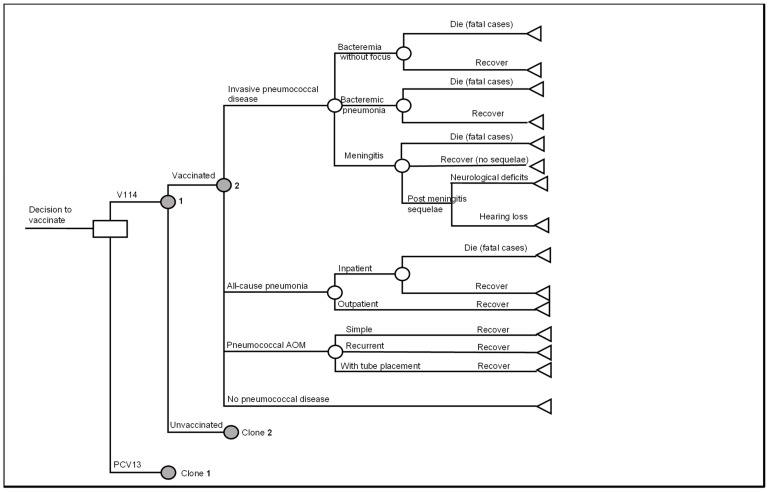
Decision analytic model of pediatric pneumococcal vaccination. Abbreviations: PCV13 = 13-valent pneumococcal conjugate vaccine; V114 = 15-valent pneumococcal conjugate vaccine; AOM = acute otitis media. Notes: (1) The clone nodes are identical in structure to the original nodes but can have different probabilities of events. (2) general background mortality applies to all health states.

A decision analytic model with a Markov structure was developed in Microsoft^®^ Excel 365 (Microsoft Corporation, Redmon, WA, USA) to assess the cost-effectiveness of routine pediatric vaccination with V114 versus PCV13 in the 3 + 1 dosing schedule in the US. (Figure 1). In the model, all three doses in the primary series occurred in the first year of life and the booster dose occurred in the second year of life. The primary series and the booster were the same type of vaccine, i.e., all V114 or all PCV13. Mixed use of PCV13 and V114 was not considered in the model.

Although the vaccination program targets infants and toddlers, the target population of the model included the entire US population, taking into account the herd immunity effects on vaccinated and non-vaccinated individuals. The model was designed to follow the current US population from age 0 to 100 years old and allow new birth cohorts to enter the population each year with a one-year cycle length. The base-case model adopted a lifetime horizon (i.e., 100 years) and applied a societal perspective, which considered direct medical, direct non-medical and indirect costs. The incremental cost-effectiveness ratio (ICER), expressed as cost per life year (LY) and cost per quality-adjusted life-year (QALY) gained, was evaluated. In addition, clinical outcomes, including the number of IPD, all-cause pneumonia, pneumococcal AOM, post-meningitis sequelae (PMS) cases prevented, and IPD-related and pneumonia-related deaths avoided, were reported. All-cause pneumonia was included as an outcome due to a lack of robust data on pneumococcal pneumonia. Costs, QALYs and LYs were discounted using an annual discount rate of 3% [30]. Clinical outcomes such as numbers of cases or deaths were not discounted.

### 2.2. Model Structure

The Markov model consisted of three health states: no PD, PMS and death; and tracked the occurrences of acute PD events (Figure 1).

Individuals entered the model in the “no PD” health state and were subject to PD, including IPD (including meningitis, bacteremia without focus and bacteremic pneumonia), all-cause pneumonia (including inpatient and outpatient pneumonia), and pneumococcal AOM (including simple AOM, recurrent AOM and AOM with tube placement) (Figure 1). All PD events were modeled as short-term events that incurred one-time resource use and costs, as well as QALY decrement and were assumed to resolve within a year (one model cycle).

Subjects who had meningitis might develop PMS and would remain in the “PMS” state until death. They were still at risk of developing non-meningitis IPD, pneumonia and AOM in later model cycles. The model considered two categories of PMS, i.e., neurologic deficits and hearing loss [31].

Disease-related death was modeled for individuals with IPD or all-cause inpatient pneumonia, while the general background mortality was applied to all health states.

### 2.3. Model Inputs

#### 2.3.1. Target Population Size, Background Mortality and Vaccine Coverage

The birth rate and the overall population size per age group in cycle 0 (i.e., year 2022) were estimated based on the most recent US national census data [32]. Age-specific annual probabilities of death for the general population were obtained from the US National Vital Statistics Report [33] and were used as the background mortality.

In the base case, V114 and PCV13 were assumed to have the same vaccine coverage rate (VCR), which was 91.9% for the primary series and 82.4% for the booster dose based on the CDC data in 2017 [34]. The model assumed that the VCR remained constant over time. Only children who receive all 3 doses in the primary series are considered vaccinated.

#### 2.3.2. Baseline PD Incidence and Case-Fatality Rates

Table 1 presents the baseline incidence rates for different types of PDs, PMS, and the case-fatality ratios associated with IPD and inpatient pneumonia.

The IPD incidence rates stratified by age group were obtained from the CDC Active Bacterial Core (ABC) surveillance report 2018 [35]. The distribution of meningitis, bacteremia without focus and bacteremic pneumonia for IPD cases, were obtained from the same source and assumed to be the same for all age groups (Table 1). Proportions of patients with meningitis who develop PMS, including neurological deficits and hearing loss, were obtained from a systematic literature review by Jit et al. (2010) [36]. The age-specific incidence rates of all-cause inpatient and all-cause outpatient pneumonia were obtained from US claims data analysis by Tong et al. (2018) and Hu et al. (2020) [12,37]. The incidence rates of pneumococcal AOM were estimated by multiplying the incidence rates of all-cause AOM published by Hu et al. (2022) [38]. and the proportion of AOM attributable to *S. pneumoniae* reported by Kaur et al. (2022) (Table 1) [39].

Regarding mortality, the model assumed that all subjects transitioned to death at the background mortality rates of the US general population. In addition, IPD and all-cause inpatient pneumonia were associated with increased fatality rate compared to the background mortality. The case fatality rates for IPD were obtained from the studies by Olarte et al. (2015) and Moore et al. (2015) and published statistics by the CDC ABC surveillance (Table 1) [40,41,42]. The case fatality rates for all-cause inpatient pneumonia were obtained from the analysis based on the US population conducted by Hu et al. (2020) and Wuerth et al. (2016) [37,43].

#### 2.3.3. Vaccine Effectiveness

Vaccine effectiveness (VE) was modeled as a percentage reduction in baseline incidence rates of IPD, all-cause pneumonia, and pneumococcal AOM due to the administration of a pneumococcal vaccine. For IPDs, simple, and recurrent AOMs, and AOM with tube placement, serotype-specific VEs were applied; while for both inpatient and outpatient all-cause pneumonia, an overall VE was applied for each vaccine.

##### IPD

The VEs for the PCV13 serotypes were obtained from two sources (Table 2). The PCV13 VEs for the serotypes shared by PCV7 were assumed to be the same as the VE estimates for PCV7 reported by Whitney et al. (2006) [44]. The rationale for this assumption was that the CDC ABC surveillance data showed minimal increases in disease burden of the PCV7 serotypes after the introduction of PCV13 [40]. The VEs for the six serotypes covered by PCV13 but not PCV7 (i.e., 3, 6A, 19A, 1, 5 and 7F) were obtained from a case–control study by Moore et al. (2016) [45]. The serotype-specific VEs against IPD for V114 and PCV13 were assumed to be the same for the shared 13 serotypes. For the additional two serotypes in V114 (i.e., 22F and 33F), the overall PCV13 VE reported by Moore et al. (2016) [45] was applied.

##### All-Cause Pneumonia

The VEs against all-cause inpatient and outpatient pneumonia of V114 and PCV13 were derived using the VEs reported in PCV7 clinical trials adjusted based on the serotype coverage of PCV7 in the pre-PCV7 era and the serotype coverage of the newer vaccines in the most current time. The VE of PCV7 was 17.7% against all-cause inpatient pneumonia and 6% against all-cause outpatient pneumonia based on the relevant clinical trials published by Nelson et al. (2008) and Black et al. (2000) [47,50]. Based on the CDC ABC surveillance report, [40]. at the time of introduction in 2000, PCV7 covered 80.1% of the prevalent *S. pneumoniae* serotypes, while the proportion of serotypes covered by PCV13 and V114 in the post-PCV era were 24.8% and 41.6%, respectively, in 2017. For example, the VE against all-cause inpatient pneumonia for V114 was 9.2%, which was calculated as the VE of PCV7 (17.7%) multiplied by the ratio of serotype coverage between V114 and PCV7 (41.6%/80.1%) (Table 2).

##### AOM and Tube Placement

The same serotype-specific VEs against AOM episodes were applied for the serotypes shared by V114 and PCV13. For the serotypes covered by PCV7, the VE was obtained from the PCV7 clinical trial evaluating the PCV7 VE against pneumococcal vaccine serotype AOM episodes published by Eskola et al. (2001) [46]. (Table 2). For the serotypes covered by PCV13 but not PCV7, the serotype-specific VEs were based on effectiveness estimates in the US reported by Pichichero et al. (2018) [22]. The VEs against the serotypes unique to V114 were assumed to be the same as the VE against the serotypes covered by PCV7, i.e., 57% (Table 2). The serotype distribution for AOM was derived from an AOM surveillance study by Kaur et al. (2022) [39]. The same serotype-specific VEs and serotype distribution were applied in the VE estimation for both simple AOM and recurrent AOM.

The VE of PCV7 against ventilatory tube placement was 20.3%, in the clinical trial reported by Black et al. (2000) [47]. VEs of PCV13 and V114 were adjusted by the proportion of pneumococcal AOM reported by Kaur et al. (2017), [48] and for serotype coverage differences at the time of implementation. The serotype coverage was 67.1% for PCV7 serotypes in the pre-PCV7 period reported by Joloba et al. (2001), [49] and 9.3% and 17.5%, respectively, for PCV13 and V114 in the post-PCV era reported by Kaur et al. (2022) [39]. Assuming the same VE for all serotypes, the serotype specific VEs of V114 and PCV13 against pneumococcal AOM tube placement were derived (Table 2).

##### Vaccine Effect Waning

The model also accounted for the vaccine effect waning in the base case. It was assumed that vaccinated children received the full benefit of the vaccine (i.e., no waning effectiveness) during the first five years after vaccination and then the VE decreased linearly to 0% over the next 10 years (i.e., from Year 6 to Year 15 after the last vaccine dose) [51].

##### Herd Immunity on IPD

Herd immunity, also known as indirect effects of vaccines, was applied in the model as a relative reduction in the IPD incidence in the entire population. The introductions of pediatric vaccination of PCV7 and PCV13 have led to reductions in IPD in adults in the US [42,52,53]. Therefore, a similar reduction in the IPD incidence related to the two new serotypes in V114 (i.e., 22F and 33F) due to herd immunity was assumed and was incorporated into the model for the first five years following the introduction of V114 in the pediatric population.

In the base case, a reduction of 7.8% per year in 22F- and 33F-related IPD incidence rate was applied, which was assumed to be the same as the estimated indirect effect of PCV13 in a previous cost-effectiveness study by Stoecker et al. (2013) [54]. This resulted in a relative reduction of the incidence rate of 7.8%, 15.5%, 21.6%, 27.7% and 33.4% for the 1st to 5th year upon the V114 vaccine launch compared to the baseline rate. The relative reduction was assumed to remain at 33.4% after the 5th year. As the vaccination program continuously vaccinates young children, the model applied the indirect effects to all individuals (including vaccinated and unvaccinated children) throughout the entire modeled time horizon.

#### 2.3.4. Utility Inputs

Utilities for the health states were obtained from the literature. Specifically, age-specific utilities for “no PD” states were assumed to be the same as the ones in the US general population from Szende et al., (2014) [55]. Utilities for PMS, i.e., neurological deficits and hearing loss, were 0.68 and 0.73, respectively, based on a study by Rubin et al. (2010) [56] and were applied to all age groups in that state. Death had a utility of 0. In addition, QALY decrements, reported by Rubin et al. (2010) [56] and Mangen et al., (2015), [57] were applied to each episode of PD events and were assumed to be different between children <18 years and adults (Table 3).

#### 2.3.5. Cost Inputs

The base-case model adopted the societal perspective, which considered direct medical and non-medical costs as well as indirect costs. The details of cost inputs are described below. All costs are presented in 2021 US dollars (USD), and wherever applicable, the original costs were adjusted to the 2021 USD using the consumer price index for medical care [58].

##### Vaccine Acquisition and Administration Costs

The vaccine acquisition cost per dose for PCV13 was assumed to be a blended price, with 65% of the public price and 35% of the private price based on a previously published cost-effectiveness study of PCV13 [54]. Based on the PCV13 publicly contracted Vaccines for Children (VFC) price of $150.08 and the PCV13 private price (list price) of $225.68, a blended price of $176.54 was used in the base-case model (Table 4). As V114 price in the pediatric indication was unknown at time of the analysis, price parity was assumed in the base case analysis.

Vaccine administration costs, which consisted of healthcare professional costs, consumable costs, disposable costs, and practice expense costs, were obtained from the literature [59]. After adjusting for inflation, the administration cost was estimated to be $15.50 per dose for both PCV13 and V114 (Table 4).

##### Direct Medical Costs

Direct medical costs included costs related to the treatment of patients with IPD, pneumonia, AOM and PMS (Table 4). Direct medical costs for PDs was estimated for each episode of disease and they differed by PD type and age [60,61]. Direct medical costs for PMS were estimated based on the lifetime costs from the CDC report and was annualized to estimate the annual medical cost for both neurologic deficits and hearing loss.

##### Direct Non-Medical Costs and Indirect Costs

Direct non-medical costs included costs borne by patients’ families, such as non-prescription medications, special education, and transportation costs. Indirect costs included the productivity loss related to the treatment of PDs, incurred by adult patients and caregivers of pediatric patients. The direct non-medical costs and indirect costs related to productivity loss per episode were estimated by PD type and age using published studies by Stoecker et al. (2013) and McLaughlin et al. (2015) [54,62]. The direct non-medical costs for PMS were estimated using the same data source [63] and method as the ones for the direct medical costs for PMS. The annual indirect costs for PMS were derived based on the income distribution by age group in the general US population published by the Bureau of Labor Statistics [64]. The combined direct non-medical and indirect costs for PMS by age group are presented in Table 4. In addition, the model also considered indirect costs associated with productivity loss due to PD-related premature death before the life expectancy has been reached. The annual cost of premature death was estimated based on the annual earnings listed in Table 4, which was assumed to be the median earnings in 2021 for each age group, calculated by multiplying the labor force participation rate with the median weekly earnings for each age group and 52 weeks per year [64,65].

### 2.4. Sensitivity Analyses

To evaluate the uncertainties of certain model assumptions and inputs, several scenario analyses were conducted, using alternative assumptions regarding the target population, time horizon, model perspective, vaccine waning and herd immunity. Another scenario analysis was conducted using alternative input values on VEs, based on estimates from a study by Ryman et al. (2022) [66] which were generated from the post-primary series immunogenicity (IgG GMC) of V114 and predicted a higher VE against IPD and AOM on serotype 3 for V114 compared to PCV13. Additional scenario analysis explored different values for QALY decrements of PDs [67]. More details on the assumptions and model inputs used in the scenario analysis are provided in Supplementary Tables S1–S3.

In addition, one-way sensitivity analysis (SA) was conducted to evaluate the sensitivity of the model results to the values of various model inputs. The one-way SA varied the value of one parameter input at a time and illustrated the results of the most impactful parameter inputs in a tornado diagram. Moreover, a threshold analysis was conducted to determine the threshold price, which was defined as the maximum price for V114 to remain cost-saving.

Lastly, a probabilistic sensitivity analysis (PSA) was conducted to evaluate the variance of ICER when multiple inputs were varied at the same time. The inputs considered in the PSA included baseline PD incidence rates and case fatality rates, VEs of PCV13 and V114 against different PDs, indirect effects, costs, utilities of the health states and QALY decrements of PDs. A Monte Carlo simulation with 1000 iterations was conducted. Incremental costs and incremental QALYs from each iteration were illustrated in a scattered plot.

## 3. Results

### 3.1. Base Case

In the base case, the V114 vaccination strategy was a dominant strategy, with fewer PD cases, fewer PD-related deaths, longer LYs and QALYs but lower total costs, compared to the PCV13 vaccination strategy (Table 5). Specifically, over a 100-year time horizon, V114 was expected to reduce 185,711 IPD cases, 987,727 pneumonia cases, 11,151,473 AOM cases, and 3592 PMS cases compared to PCV13 vaccination strategy. It was also predicted to reduce 20,197 IPD deaths and 41 pneumonia deaths. As a result, V114 was associated with 90,026 LYs saved and incremental total QALYs of 96,056, compared to PCV13. V114 was predicted to have incremental vaccine acquisition and administration costs of $23,166 and $2034, respectively (Table 5). The differences resulted from a slightly higher eligible number of children in the second year in the V114 vaccination strategy due to the reduction in the PD-related deaths associated with V114 (versus PCV13) at the end of the first year. However, V114 was associated with lower costs in all other cost components (Table 5). In terms of direct medical costs, V114 was predicted to save $2.6 billion for IPD treatment costs, $2.2 billion for all-cause pneumonia treatment costs, $1.8 billion for pneumococcal AOM treatment costs and $155.8 million for PMS treatment costs. Adding together, V114 was associated with total direct medical cost savings of $6.8 billion. Furthermore, V114 was associated with $4.0 billion savings in the direct non-medical/indirect costs including costs of premature death. The total cost savings associated with V114 versus PCV13 were predicted to be $10.8 billion over the 100-year time horizon.

### 3.2. Sensitivity Analysis

Consistent with the base-case results, all scenario analyses showed that V114 was the dominant vaccination strategy compared to PCV13, with better clinical outcomes, longer LYs and QALYs, and lower total costs (Supplementary Table S4). One scenario evaluated the results with a single birth cohort and the assumption of full vaccine protection for the first 10 years and no VE thereafter. Under this scenario, V114 was associated with total cost savings of $213.3 million and 1329 more QALYs. Using alternative data sources for VEs against IPD and simple and recurrent AOM yielded slightly higher cost savings and greater QALY gains, with total cost savings of 11.1 billion and 98,501 more QALYs for the V114 strategy. Applying different values for QALY decrements of PDs led to QALY gains of 85, 926 (compared to 96, 056 in the base case analysis). Moreover, the scenario analysis assuming no herd immunity resulted in total cost savings of 5.7 billion and 28,050 more QALYs in the V114 strategy. In all other scenarios, the total cost savings ranged from $175.0 million to $17.2 billion, and the incremental QALYs ranged from 916 to 181,102.

**Figure 2:**
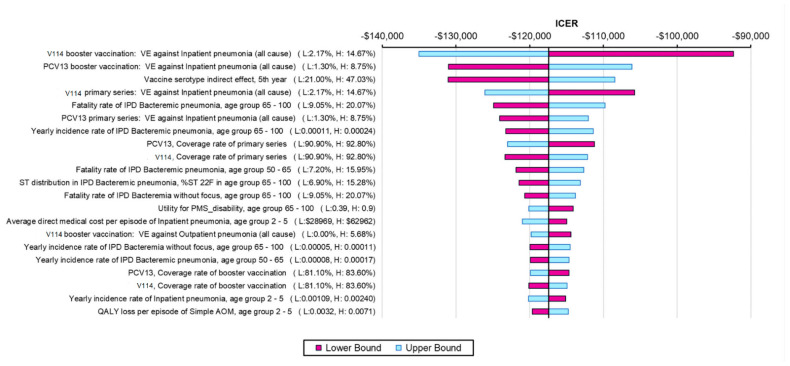
One-way sensitivity analysis results (showing the most impactful parameters). Abbreviations: ICER = incremental cost-effectiveness ratio; PCV13 = 13-valent pneumococcal conjugate vaccine; V114 = 15-valent pneumococcal conjugate vaccine; VE = vaccine effectiveness; IPD = invasive pneumococcal disease; AOM = acute otitis media; ST = serotype; QALY = quality-adjusted life year. Notes: Tornado diagram showing the results of the one-way sensitivity analysis. The purple bars show the change in ICER from the base case when the higher value of an input was used whereas the light blue bars show the change in ICER from the base case when the lower value of the selected input was used while all other inputs remain constant.

**Figure 3:**
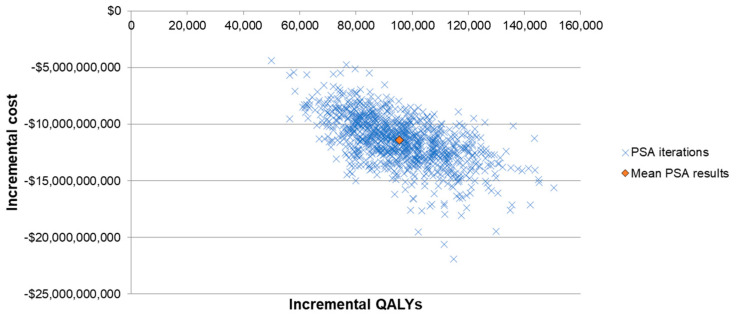
Scattered plot of probabilistic sensitivity analysis. Abbreviations: QALY = quality-adjusted live year; PSA = probabilistic sensitivity analysis.

The results from the one-way SA further confirmed the robustness of the model results, with all ICERs showing that V114 was a dominant strategy (Figure 2). The ICER was most sensitive to VEs against all-cause inpatient pneumonia, VCR, indirect effects and incidence and fatality rates of bacteremic pneumonia in the elderly (due to the high incidence and fatality of the disease in this age group). The threshold price per dose for V114 was $28 higher than PCV13′s price. The results from the PSA showed that 100% of the ICERs were in the fourth quadrant in the scattered plot, indicating more QALYs and lower costs for the V114 strategy (Figure 3).

## 4. Discussion

To our knowledge, this is the first study evaluating the cost-effectiveness of V114 as a national pediatric vaccination program. The findings suggest that V114 is a dominant strategy compared to PCV13, with substantial cost savings and better clinical outcomes and QALYs.

Over 100 years after the introduction of V114, vaccinating 345 million infants with V114 instead of PCV13 would reduce 185,711 IPD cases, 987,727 all-cause pneumonia cases, 11 million pneumococcal AOM cases, and over 20,000 PD-related deaths in the US population. V114 is predicted to extend the total LYs by 90,026 and total QALYs by 96,056, compared to PCV13. In addition, it is associated with total cost savings of $10.8 billion from the societal perspective, of which $6.8 billion are attributable to direct medical cost savings. Thus, V114 is a cost-saving strategy not only from the societal perspective but also from the health-care sector perspective. All three types of PDs contributed significantly to the direct medical cost savings, but IPD treatment costs contribute most ($2.6 billion).

V114 remained a dominant strategy compared to PCV13 in all scenario analyses and one-way SAs explored. It is also predicted to be 100% dominant versus PCV13 in the PSA. Therefore, the findings from the current study are robust under the alternative assumptions and the uncertainties of model inputs. The model results are mostly impacted by the target population, time horizon and assumptions/inputs related to the indirect effects. Both direct and indirect effects of vaccination contribute to the cost savings and QALY gains. The indirect effects accounted for approximately 47% of the total cost savings and 67% of the QALY gains in the entire population. Based on PCV7/13 precedent [42,52,53,68,69,70] indirect effects are an important and legitimate consideration when estimating the outcomes of pneumococcal vaccine, and as such their exclusion would represent an extreme and unrealistic scenario. Similar to the findings of this current study, previous studies demonstrated that indirect effects have a substantial impact on the cost-effectiveness of pneumococcal vaccines and the results are sensitive to the magnitude and duration of the indirect effects. For example, a cost-effectiveness analysis of PCV7 showed that after accounting for the indirect effects, the number of IPD cases averted increased from 38,000 to 109,000 and the ICER reduced from $102,000 per LY saved to $7500 per LY saved in a 5-year time horizon [59]. Similarly, a UK analysis of PCV7 showed that the ICER in cost per LY saved reduced by 87% after incorporating the indirect effect in the model and the ICER was sensitive to the magnitude of the indirect effects [71]. Another cost-effectiveness analysis of PCV13 versus PCV7 demonstrated an 88% reduction in QALYs and 97% reduction in cost savings per child vaccinated after removing the indirect effects [56]. Despite the sensitivity of model results to the assumptions of indirect effects, the current study still showed that V114 remained a dominant strategy when excluding indirect effects. In addition, V114 was shown to be cost saving even if the unit price was $28 higher than PCV13.

The findings from this study are also consistent with the previous studies evaluating the cost-effectiveness of a pneumococcal vaccine. PCV7 has been consistently shown to be cost-effective versus no vaccination in the US, in spite of higher total costs due to the costs associated with the vaccination program [59,72,73]. Several studies that assessed the cost-effectiveness of PCV13 versus PCV7 or PCV10 demonstrated that PCV13 was a dominant strategy or cost-effective in all base-case analyses and scenario analyses [56,74,75,76,77]. For example, using a similar model structure to our study, Rubin et al. predicted that PCV13 would prevent 106,000 IPD cases and save $11.6 billion compared to PCV7 in the US over a 10-year time horizon [56].

Since the implementation of PCVs in the pediatric national immunization program, there has been a shift to NVT IPD. A meta-analysis showed that 57.8% of the pediatric IPD cases were caused by non-PCV13 serotypes in North America in post-PCV13 era (between 2010 and 2015), among which approximately 20% were attributable to 22F and 33F, the two serotypes covered by V114 but not PCV13 [21]. More recent US studies showed a similar proportion (approximately 22%) of 22F and 33F among non-PCV13 serotypes [19,26] but a higher proportion of IPD cases among children <5 years caused by non-PCV13 serotypes (77%) in 2017–2018 [26]. In addition, PCV13 has limited VE against serotype 3, which represents ~6% of the IPD cases in North America in post-PCV13 era [21]. With superior immune response against serotypes 3, 22F and 33F, [29]. V114 can effectively protect against the disease-causing vaccine-type serotype and the two most prevalent NVT serotypes. As a result, V114 is expected to substantially reduce the costs associated with PDs. The robust evidence on the clinical and economic benefits of V114 supports its inclusion in the pediatric vaccination program in the US.

There are several limitations associated with the current study. First, Markov models are static, simplified models that are not always suitable to model transmission dynamics in infectious diseases. However, this concern is somewhat mitigated because the model applied an alternative approach to account for the indirect effects associated with V114 and evaluated different assumptions and inputs related to indirect effects. Regarding that, the model applied a relatively conservative assumption of indirect effects, which were assumed to be applicable only to IPD for a period up to five years, while indirect effects may extend to other types of PDs, including all-cause pneumonia and AOM based on the existing evidence [69,70]. On the other hand, the model did not account for the fact that V114 and PCV20 are being used in the adult population, which may reduce the indirect effects. However, this does not change the conclusion of the current study as V114 remained a dominant strategy after removing the indirect effects. In addition, due to a lack of reliable data, several assumptions were made regarding the effectiveness of V114 and PCV13. For example, the VEs were based on PCV7 data adjusted by the serotype coverage. When the VE for a certain serotype is missing, it was imputed with the average VE against other serotypes. These estimations or assumptions introduced uncertainties about the VEs of the two vaccination strategies. Even though the model results were sensitive to certain VEs, the conclusion that V114 is cost saving remains robust in all sensitivity analyses. Additionally, in a Phase 3 clinical trial V114 demonstrated a superior immune response against serotype 3 compared to PCV13 [28]. The model base case, which assumed the two vaccines had the same VEs against all PCV13-type diseases, might lead to underestimation of the benefit of V114. To address this, a scenario analysis was conducted using the VE estimates derived from the immunogenicity data. However, because of high level of uncertainty around these VE estimates, further research and more robust data are important to quantify the impact of superior immune response of V114 on serotype 3. Furthermore, the current study assumed that the serotype distribution remained static over the modeled time horizon. Recent studies demonstrated that using time-varying serotype epidemiology has a big impact on the results of a model with a multi-cohort target population [78,79]. However, recent data suggest that serotype replacement is not evident in the US in the post-PCV13 era [80,81]. In addition, the model applied vaccine effect waning assumptions, which may partially account for the potential changes in serotype distribution. Given the robustness of the model’s results, it is not expected that lack of explicit modeling of time-varying serotype distribution will change the conclusion of the current study. Finally, the model did not account for certain types of costs. For example, because the primary treatment for PDs consists of antibiotics, there is a high potential for the development of pneumococcal antibiotic resistance over time [82], which may lead to treatment failure and increased treatment costs for PDs. This current analysis did not include the associated health and economic impacts of antibiotic resistance, which is a conservative approach likely to have underestimated savings; it is expected that additional cost savings would be afforded by V114 due to prevention of resistant PD cases since treatment costs for resistant cases are higher [83]. As another example, the current model did not include non-medical and indirect costs related to vaccination. It is expected this has minimal impact on the model results because the vaccinations normally occur during routinely scheduled well-child visits for infants and toddlers, and the total number of vaccinations was similar between V114 and PCV13 strategies.

Despite these limitations, the current study applies a well-established model structure for pneumococcal vaccines in the evaluation of the cost-effectiveness of V114 versus PCV13. The current model approach is improved from previous analyses given the application of serotype-specific VEs for IPD and simple and recurrent AOM, which allows serotype-specific health and economic impact assessment of these PDs. In addition, the model also incorporated the most recent data, e.g., the baseline incidence rates of all-cause pneumonia and pneumococcal AOM, and current costs associated with PDs. As such, these data are more relevant to current decision making. Furthermore, extensive scenario analysis, one-way SAs and PSA were conducted in the current study, and the cost-saving results for V114 are robust under all plausible alternative assumptions and a wide range of model input values.

## 5. Conclusions

The study suggests that routine pediatric vaccination with V114 prevents a considerable number of PD cases versus PCV13, which results in extended LYs and QALYs in the US population, and substantial cost savings from both direct and indirect costs associated with PDs. The findings are robust under all plausible alternative assumptions and a wide range of model input values. The study further supports the ACIP’s recommendations for V114 to be included as an alternative pediatric pneumococcal vaccination strategy in the US.

## Figures and Tables

**Table 1:** Pneumococcal disease incidence and case fatality rates by age.

Parameter	Age Group (Years)									
	<1	1	2–4	5–17	18–34	35–49	50–64	65–74	75–84	85+
Annual incidence rates (per 100, 000)										
IPD ^a^	13.3	9.5	4.3	1.7	2.8	7.1	16.6	24.0	24	24
% IPD that is meningitis ^a^	7.2									
% IPD that is bacteremia without focus ^a^	21.4									
% IPD that is bacteremic pneumonia ^a^	71.4									
Inpatient pneumonia (All-cause) ^b,c^	339	339	168	45	58	58	204	723	1852	3694
Outpatient pneumonia (All-cause) ^b,c^	2906	2906	3413	1333	622	622	1106	1917	3408	5876
Simple AOM (All-cause) ^d^	54,200	54,200	33,800	8400	-	-	-	-	-	-
Recurrent AOM (All-cause) ^d^	19,600	19,600	6900	500	-	-	-	-	-	-
AOM tube placement (All-cause) ^d^	4340	4340	1740	200	-	-	-	-	-	-
% AOM and tube placement attributable to *S. pneumoniae* ^e^	23.8									
Case fatality rates (%)										
Meningitis ^f, h^	10.00	10.00	10.00	10.00	6.90	7.30	11.20	14.11	14.11	14.11
Bacteremia without focus/bacteremic pneumonia ^g, h^	3.00	3.00	3.00	3.00	6.90	7.30	11.20	14.11	14.11	14.11
Inpatient pneumonia (All-cause) ^i, j^	0.29	0.29	0.29	0.51	1.7/2.4 *	3.5/4.9 *	4.9/5.5 *	6.9	8.2	11.6/15.4 *
PMS ^k^										
% meningitis cases leading to neurological deficits	12.2	12.2	12.2	12.2	31.7	31.7	31.7	31.7	31.7	31.7
% meningitis cases leading to hearing loss	8.2	8.2	8.2	8.2	-	-	-	-	-	-

Abbreviations: IPD = invasive pneumococcal disease; AOM = acute otitis media; PMS = post meningitis sequelae. Notes: a—The Centers for Disease Control and Prevention Active Bacterial Core (CDC ABC) surveillance report 2018 [35]; b—The data source for pediatric (<18 years) all-cause pneumonia incidence was based on an analysis of the MarketScan commercial claims database from 2018 [37]. c—The incidence rates of age stratified all-cause inpatient and outpatient pneumonia in adults were obtained from Tong et al., 2018 for the year 2014 [12]. d—The incidence rates of all-cause simple AOM, recurrent AOM and AOM tube placement in children <18 years were estimated using the MarketScan commercial claims database for 2018 [38]. e—Based on the serotyping of middle ear fluid collected from children with AOM between 2015 and 2019 by Kaur et al., 2022 [39]. f—The case fatality ratios (CFR) for meningitis for children <18 years were obtained from Olarte et al., 2015 [41] g—The CFR of bacteremia without focus and bacteremic pneumonia for children <18 years were from Moore et al., 2015 [42]. h—The CFR of IPD for the adult population were from CDC ABC surveillance data [40]. i—Case fatality rates for children <18 years of age are derived from Hu et al., 2018 [37]. j—Case fatality rates for individuals over 18 years old are from Wuerth et al., 2016 [43]. *—Case fatality rates of inpatient pneumonia are 1.7% (in age group 18–24), 2.4% (25–34), 3.5% (35–44), 4.9% (45–54), 5.5% (55–65), 11.6 (85–94), and 15.4% (95+). k—Based on a systematic literature review conducted by Jit et al., 2010 [36].

**Table 2:** Vaccine effectiveness of PCV13 and V114 in the base case.

**Disease**	**Vaccine Type**	**Serotype-Specific Effectiveness**														
	**Serotype**	**1**	**4**	**5**	**6B**	**7F**	**9V**	**14**	**18C**	**19F**	**23F**	**3**	**6A**	**19A**	**22F**	**33F**
**IPD ^a^**	PCV13	87%	93%	87%	94%	97%	100%	94%	97%	87%	98%	80%	86%	86%	-	-
	V114	87%	93%	87%	94%	97%	100%	94%	97%	87%	98%	80%	86%	86%	86%	86%
**AOM ^b^ (pneumococcal)**	PCV13	86%	57%	86%	57%	86%	57%	57%	57%	57%	57%	15%	100%	91%	-	-
	V114	86%	57%	86%	57%	86%	57%	57%	57%	57%	57%	15%	100%	91%	57%	57%
**AOM** **tube placement ^c^ (pneumococcal)**	PCV13	69%	69%	69%	69%	69%	69%	69%	69%	69%	69%	69%	69%	69%	-	-
	V114	69%	69%	69%	69%	69%	69%	69%	69%	69%	69%	69%	69%	69%	69%	69%
**Disease**							**Vaccine type**				**Effectiveness**					
**Inpatient pneumonia ^d^ (all-cause)**							PCV13				5.5%					
							V114				9.2%					
**Outpatient pneumonia ^d^ (all-cause)**							PCV13				1.9%					
							V114				3.1%					

Abbreviations: PCV13 = 13-valent pneumococcal conjugate vaccine; V114 = 15-valent pneumococcal conjugate vaccine; PD = pneumococcal disease; IPD = invasive pneumococcal disease; AOM = acute otitis media. Notes: a—Vaccine effectiveness (VE) for IPD: Whitney et al., 2006; [44] Moore et al., 2016 [45]. b—VE for simple and recurrent AOM: Eskola et al., 2001; [46] Pichichero 2018 [22]. c—VE for tube placement: Black et al., 2000; [47] adjusted by the proportion of pneumococcal AOM (Kaur et al., 2017 [48]), and serotype coverage (Joloba 2001 [49] and Kaur 2022 [39]). d—VE for all-cause pneumonia: Nelson et al., 2008; [50] Black et al., 2000; [47] adjusted by the ratio of the age-specific serotypes covered by PCV13 in 2017 and by PCV7 in 2000 [40].

**Table 3:** Health state utility values and QALY decrements of PD events.

**Parameter**	**Age Group (Years)**							
	**<18**	**18–24**	**25–34**	**35–44**	**45–54**	**55–64**	**65–74**	**75+**
**Health State Utility Values**								
Baseline (general population without PD) ^a^	0.92	0.92	0.91	0.89	0.85	0.83	0.81	0.75
PMS, neurological deficits ^b^	0.68							
PMS, hearing loss ^b^	0.73							
**QALY decrements related to each episode of PD events ^c^**	**Age group (years)**							
	**<18**	**18+**						
Meningitis	0.023	0.071						
Bacteremia without focus/bacteremic pneumonia	0.008	0.071						
Inpatient pneumonia (All-cause)	0.006	0.071						
Outpatient pneumonia (All-cause)	0.004	0.005						
AOM and tube placement	0.005	-						

Abbreviations: QALY = Quality-adjusted life year; PD = pneumococcal disease; PMS = post meningitis sequelae; AOM = acute otitis media. Notes: a—The baseline utility values for the individuals without PD in the US were reported by Szende et al., 2014 [55]. b—Health state utilities for PMS were obtained from the study by Rubin et al., 2010 [56]. c—For children under 18 years old, the QALY decrements of IPD and all-cause pneumonia, and AOM were obtained from the study by Rubin et al., 2010 [56] For patients 18 years and older, the QALY decrements were obtained from the study of Mangen et al., 2015 [57].

**Table 4:** Cost inputs.

**Cost Component**	**Input Value**								
**Vaccine Costs (in 2021 USD)**									
Vaccine acquisition costs (per dose) ^a^									
PCV13	176.54								
V114	176.54								
Vaccine administration costs (per dose) ^b^									
PCV13	15.5								
V114	15.5								
**Costs per episode of PD events (in 2021 USD)**	**Age group (years)**								
	**<2**	**2–4**	**5–17**	**18–34**	**35–49**	**50–64**	**65–74**	**75–84**	**85+**
Direct medical costs ^c^									
Meningitis	65,419	65,419	65,419	57,657	57,657	58,890	28,217	28,217	28,217
Bacteremia without focus	46,909	46,909	46,909	57,657	57,657	58,890	28,217	28,217	28,217
Bacteremic pneumonia	58,774	58,774	58,774	57,657	57,657	58,890	28,217	28,217	28,217
Inpatient pneumonia (All-cause)	42,708	42,708	42,708	25,814	25,814	27,797	18,400	18,400	18,400
Outpatient pneumonia (All-cause)	525	525	525	812	812	774	671	671	671
Simple AOM	291	291	291	-	-	-	-	-	-
Recurrent AOM	711	711	711	-	-	-	-	-	-
AOM tube placement	2653	2653	2653	-	-	-	-	-	-
Direct non-medical and indirect costs ^d^									
Meningitis	3416	983	983	3345	3345	3345	1342	892	806
Bacteremia without focus	652	983	983	3345	3345	3345	1342	892	806
Bacteremic pneumonia	652	983	983	3345	3345	3345	1342	892	806
Inpatient pneumonia (All-cause)	487	983	983	2702	2702	2702	1084	720	652
Outpatient pneumonia (All-cause)	487	487	487	1286	1286	1286	517	344	310
Simple AOM	193	193	193	-	-	-	-	-	-
Recurrent AOM	193	193	193	-	-	-	-	-	-
AOM tube placement	482	482	482	-	-	-	-	-	-
**Annual costs of PMS and premature death (in 2021 USD)**	**Age group (years)**								
	**<16**	**16–19**	**20–24**	**25–34**	**35–44**	**45–54**	**55 -64**	**64–74**	**75+**
Direct medical costs ^e^									
PMS, neurological deficits	8116								
PMS, hearing loss	1521								
Direct non-medical and indirect costs									
PMS, neurological deficits ^f^	6820	31,850	66,069	106,986	125,566	125,838	107,502	48,166	21,937
PMS, hearing loss ^f^	8335	16,908	28,628	42,641	49,005	49,098	42,818	22,496	13,513
Annual earnings ^g^	-	10,154	24,036	40,635	48,172	48,283	40,844	16,773	6133

Abbreviations: PCV13 = 13-valent pneumococcal conjugate vaccine; V114 = 15-valent pneumococcal conjugate vaccine; PD = pneumococcal disease; AOM = acute otitis media; PMS = post meningitis sequelae. Notes: a—The vaccine acquisition costs were assumed to be a blended price for PCV13, with weights for the public and private prices of 65% and 35%, respectively, based on the study by Stoecker et al., 2013 [54]. Price parity was assumed for V114. b—Vaccine administration costs were obtained from the study by Ray et al., 2006 [59]. c—Costs for children < 18 years old were obtained from an unpublished analysis of MarketScan commercial claims database [60] For adults, the costs were obtained from the 2021 Advisory Committee on Immunization Practices (ACIP) meeting presentation by Stoecker C [61] The costs for the adult population stratified by risk group were combined using weights for proportion of each risk group in the overall population within the age strata. d—Direct non-medical costs and indirect costs related to PDs were based on the studies by McLaughlin et al., 2015 [62] and Stoecker et al., 2013 [54]. e—Direct medical costs related to PMS were based on the Morbidity and Mortality Weekly Report (MMWR) report [63]. The discounted lifetime cost was then annualized to estimate the annual medical cost for both neurologic deficits and hearing loss. f—Direct non-medical costs and indirect costs related to PMS were based on the MMWR report [63] The discounted lifetime non-medical cost was annualized to estimate the annual non-medical cost; while annual indirect costs were derived based on the income distribution by age groups in the general US population. g—Annual cost of premature death was assumed to be the same as the median annual earnings in 2021 for each age group, obtained from the Bureau of Labor Statistics (BLS) [64,65].

**Table 5:** Base-case results.

Outcomes	V114	PCV13	Incremental Outcomes (V114 versus PCV13)
**Clinical outcomes (undiscounted)**			
IPD cases	3,383,921	3,569,631	−185,711
All-cause pneumonia cases	580,425,873	581,413,600	−987,727
Pneumococcal AOM cases	289,559,830	300,711,303	−11,151,473
PMS cases	68,141	71,733	−3592
IPD deaths	391,677	411,874	−20,197
Pneumonia deaths	11,004,703	11,004,744	−41
**LYs and QALYs (discounted)**			
Total LYs	10,042,394,010	10,042,303,984	90,026
**Total QALYs**	**8,703,494,217**	**8,703,398,161**	**96,056**
**Cost outcomes (2021 USD, discounted)**			
Vaccine acquisition costs	$68,689,837,125	$68,689,813,959	$23,166
Vaccine administration costs	$6,030,885,213	$6,030,883,179	$2034
IPD treatment costs	$49,825,494,002	$52,463,715,243	−$2,638,221,241
All-cause pneumonia treatment costs	$979,125,959,933	$981,375,224,621	−$2,249,264,688
AOM treatment costs	$44,096,603,184	$45,853,332,383	−$1,756,729,199
PMS treatment costs	$3,035,895,936	$3,191,739,537	−$155,843,601
Direct non-medical/indirect costs	208,629,466,353	$210,638,743,324	−$2,009,276,972
Costs of premature death	470,698,119,691	$472,706,362,297	−$2,008,242,605
**Total Costs**	**$1,830,132,261,437**	**$1,840,949,814,543**	**−$10,817,553,106**
**ICER**			**V114 dominant**

Abbreviations: PCV13 = 13-valent pneumococcal conjugate vaccine; V114 = 15-valent pneumococcal conjugate vaccine. IPD = invasive pneumococcal disease; AOM = acute otitis media; PMS = post meningitis sequelae; LY = life year; QALY = quality-adjusted life year; ICER = incremental cost-effectiveness ratio.

## Data Availability

The data used in this study are presented in the article or the Supplementary Materials.

## Supplementary Materials

The following supporting information can be downloaded at: https://www.mdpi.com/article/10.3390/vaccines11010135/s1. Table S1: Description of the assumptions and inputs in the scenario analysis; Table S2: VE of PCV13 and V114 against IPD and AOM in the scenario analysis; Table S3: QALY decrements for IPD, pneumonia and AOM in the scenario analysis; Table S4: Incremental costs and effectiveness outcomes comparing V114 vs. PCV13 in the scenario analysis.
