# Supplementary material for: Cost-Effectiveness Analysis of Routine Use of 15-Valent Pneumococcal Conjugate Vaccine in the US Pediatric Population
Source: Vaccines (Basel). 2023 Jan 6;11(1):135. doi: 10.3390/vaccines11010135 (PMC9861214; doi:10.3390/vaccines11010135)
Supplement: Supplementary file 1 [file vaccines-11-00135-s001.zip › vaccines-2050851-supplementary.pdf]

Supplementary Table S1. Description of the assumptions and inputs in the scenario analysis

Scenario Analysis	Base-case assumptions/inputs description	Scenario analysis assumptions/inputs description
1	Target population: entire US population;<br>Vaccine waning: full vaccine protection for first 5 years after vaccination and decreased to 0 for the next 10 years	Target population: a single birth cohort<br>Vaccine waning: full vaccine protection for first 10 years after vaccination and no VE thereafter
2	Perspective: societal perspective (including vaccine acquisition and administration costs, direct medical costs, direct non-medical costs, indirect costs and costs of premature death)	Perspective: health care sector perspective (only including vaccine acquisition and administration costs and direct medical costs)
3	Time horizon: 100 years (lifetime)	Time horizon: 10 years
4	VEs against IPD, simple and recurrent AOM: Table 2	VE against IPD, simple and recurrent AOM: Supplementary Table 3 (Ryman et al. 2022, generated from the post-primary series immunogenicity (IgG GMC) of V114)[a]
5	Inputs of QALY decrements related to each PD episode: Table 3 (Rubin et al., 2010 and Mangen et al., 2015)[b,c]	Inputs of QALY decrements related to each PD episode: Supplementary Table 4 (Tang et al., 2022)[e]
6	Herd immunity on IPD was applied	No herd immunity was assumed
7	Target population: entire US population;<br>Herd immunity was applied	Target population: a single birth cohort;<br>No herd immunity was assumed
8	Target population: entire US population;<br>Inputs of QALY decrements related to each PD episode: Table 3 (Rubin et al., 2010 and Mangen et al., 2015) [b,c]	Target population: a single birth cohort;<br>Inputs of QALY decrements related to each PD episode: Supplementary Table 4 (Tang et al., 2022) [e]

Abbreviations: VE = vaccine effectiveness; IPD = invasive pneumococcal disease; AOM = acute otitis media; IgG=Immunoglobulin G; GMC= Geometric mean concentration (mcg/mL); V114 = 15-valent pneumococcal conjugate vaccine; QALY=quality-adjusted life years; PD = pneumococcal disease.

Notes:

a. Source: Ryman J, Weaver J, Yee KL, Sachs JR. Predicting effectiveness of the V114 vaccine against invasive pneumococcal disease in children. *Expert Rev Vaccines.* Oct 2022;21(10):1515-1521. doi:10.1080/14760584.2022.2112179
b. Source: Rubin JL, McGarry LJ, Strutton DR, et al. Public health and economic impact of the 13-valent pneumococcal conjugate vaccine (PCV13) in the United States. *Vaccine.* Nov 10 2010;28(48):7634-43. doi:10.1016/j.vaccine.2010.09.049
c. Source: Mangen MJ, Rozenbaum MH, Huijts SM, et al. Cost-effectiveness of adult pneumococcal conjugate vaccination in the Netherlands. *Eur Respir J.* Nov 2015;46(5):1407-16. doi:10.1183/13993003.00325-2015
d. Source: Tang Z, Matanock A, Jeon S, Leidner AJ. A review of health-related quality of life associated with pneumococcal disease: pooled estimates by age and type of disease. *J Public Health (Oxf).* Jun 2022;44(2):e234-e240. doi:10.1093/pubmed/fdab159

Supplementary Table s2. VE of PCV13 and V114 against IPD and AOM in the scenario analysis

Disease	Vaccine type	Serotype-specific effectiveness														
	**Serotype**	**1**	**4**	**5**	**6B**	**7F**	**9V**	**14**	**18C**	**19F**	**23F**	**3**	**6A**	**19A**	**22F**	**33F**
**IPD**	PCV13	87%	93%	87%	94%	97%	100%	94%	97%	87%	98%	30%	86%	86%	-	-
	V114	76%	95%	84%	86%	93%	99%	91%	94%	99%	99%	54%	59%	78%	86%	86%
**AOM (pneumococcal)**	PCV13	86%	57%	86%	57%	86%	57%	57%	57%	57%	57%	15%	100%	91%	-	-
	V114	86%	57%	86%	57%	86%	57%	57%	57%	57%	57%	27%	83%	91%	57%	57%

Abbreviations: VE = vaccine effectiveness; IPD = invasive pneumococcal disease; AOM = acute otitis media; PCV13 = 13-valent pneumococcal conjugate vaccine; V114 = 15-valent pneumococcal conjugate vaccine.

Supplementary Table s3. QALY decrements for IPD, pneumonia and AOM in the scenario analysis

Age group (years)	QALY decrements for IPD			QALY decrements for pneumonia		QALY decrements for AOMs		
	**Meningitis**	**Bacteremia without focus**	**Bacteremic pneumonia**	**Inpatient**	**Outpatient**	**Simple AOM**	**Recurrent AOM**	**AOM with tube placement**
0 – 17	0.0165	0.0016	0.0016	0.0105	0.0004	0.0016	0.0016	0.0016
≥18	0.0547	0.0547	0.0547	0.0396	0.0094	—	—	—

Abbreviations: QALY=quality-adjusted life years; IPD = invasive pneumococcal disease; AOM = acute otitis media.

Supplementary Table s4. Incremental costs and effectiveness outcomes comparing V114 vs. PCV13 in the scenario analysis

Scenarios	Incremental Outcomes (V114 vs. PCV13)							
	1	2	3	4	5	6	7	8
**Clinical outcomes (undiscounted)**								
IPD cases	-1,786	-185,711	-17,774	-190,699	-185,711	-22,122	-220	-1,783
Pneumonia cases	-11,315	-987,727	-81,655	-987,621	-987,727	-1,001,832	-11,169	-11,012
AOM cases	-118,497	-11,151,473	-1,051,896	-11,991,523	-11,151,473	-11,151,511	-116,752	-116,751
PMS cases	-35	-3,592	-310	-3,657	-3,592	-290	-3	-35
IPD deaths	-195	-20,197	-1,699	-20,368	-20,197	-758	-9	-195
Pneumonia deaths	-1	-41	-35	-40	-41	-513	-6	-1
**LYs and QALYs (discounted)**								
Total LYs	874	90,026	6,047	91,450	90,026	10,871	410	867
**Total QALYs**	1,329	96,056	10,696	98,501	85,926	28,050	916	958
**Cost outcomes (million 2021 USD, discounted)**								

Vaccine acquisition costs	$761	$23,166	$6,753	$25,435	$23,166	$21,785	$749	$761
Vaccine administration costs	$67	$2,034	$593	$2,233	$2,034	$1,913	$66	$67
IPD treatment costs	-$25.4	-$2,638.2	-$669.6	-$2,724.2	-$2,638.2	-$389.2	-$11.1	-$25.2
Pneumonia treatment costs	-$70.2	-$2,249.3	-$558.3	-$2,249.2	-$2,249.3	-$2,272.4	-$69.1	-$69.0
AOM treatment costs	-$53.0	-$1,756.7	-$455.4	-$1,856.2	-$1,756.7	-$1,756.7	-$52.4	-$52.4
PMS treatment costs	-$1.7	-$155.8	-$10.0	-$160.3	-$155.8	-$20.5	-$0.8	-$1.7
Direct non-medical/indirect costs	-$40.6	$0.0	-$322.2	-$2,087.7	-$2,009.3	-$1,018.9	-$32.1	-$40.1
Costs of premature death	-$22.3	$0.0	-$130.3	-$2,040.8	-$2,008.2	-$242.0	-$9.6	-$22.2
**Total Costs**	-$213.3	-$6,800.0	-$2,145.6	-$11,118.4	-$10,817.6	-$5,699.6	-$175.0	-$210.6
**ICER**	V114 dominant	V114 dominant	V114 dominant	V114 dominant	V114 dominant	V114 dominant	V114 dominant	V114 dominant

Abbreviations: PCV13 = 13-valent pneumococcal conjugate vaccine; V114 = 15-valent pneumococcal conjugate vaccine; IPD = invasive pneumococcal disease; AOM = acute otitis media; PMS = post meningitis sequelae; LY = life year; QALY = quality-adjusted life year; ICER = incremental cost-effectiveness ratio.
